# Spin-Polarized DFT+*U* Study of Surface-Functionalized Cr_3_C_2_ MXenes: Tunable Electronic and Magnetic Behavior for Spintronics

**DOI:** 10.3390/ma18153709

**Published:** 2025-08-07

**Authors:** Zixiang Tong, Yange Suo, Shaozheng Zhang, Jianhui Yang

**Affiliations:** 1 School of Mechanical and Energy Engineering, Zhejiang University of Science and Technology, Liuhe Road 318#, Hangzhou 310023, Chinasuoyange@zust.edu.cn (Y.S.); 2 College of Teacher Education, Quzhou University, Quzhou 324000, China

**Keywords:** Cr_3_C_2_ MXene, two-dimensional materials, DFT, magnetism, Curie temperature

## Abstract

Surface functionalization is key for tuning the electronic and magnetic properties essential in spintronics, yet its impact on chromium-based MXenes (Cr_3_C_2_T_2_) is not fully understood. Using spin-polarized DFT+*U*, this study investigates how O, F, and OH groups modify the magnetic state, electronic structure, and Curie temperature. Functionalization dramatically changes magnetism: O termination gives ferromagnetism, while F and OH yield ferrimagnetism. Our results show surface functionalization effectively adjusts the Curie temperature, critical for spintronic materials. The electronic character is highly functional group dependent: pristine Cr_3_C_2_ is half-metallic, Cr_3_C_2_O_2_ metallic, and Cr_3_C_2_F_2_/Cr_3_C_2_(OH)_2_ semiconducting with narrow gaps. Structures with dynamic stability are analyzed through phonon spectroscopy. These findings provide fundamental insights into controlling MXene properties via surface functionalization, guiding the design of next-generation spintronic materials.

## 1. Introduction

MXenes represent an emerging class of two-dimensional (2D) materials composed of early transition metal carbides and nitrides. Since their initial discovery by Gogotsi et al. in 2012 [1,2], MXenes have garnered substantial interest within the materials science community due to their versatile physicochemical properties and potential applications [3]. These materials are typically represented by the formula M_n+1_X_n_T_x_, where M represents a transition metal (e.g., titanium, chromium, or vanadium), X represents carbon or nitrogen, and T represents surface functional groups (e.g., O, F, or OH) [4]. The unique layered structure of MXenes imparts exceptional 2D electronic properties, such as high electrical conductivity and chemically active surfaces, making them promising candidates for a wide range of applications, encompassing energy storage [5,6], catalysis [7], sensors [8], supercapacitors [6], and next-generation electronic devices [9]. MXenes distinguish themselves from conventional 2D materials, such as graphene, MoS_2_, or h-BN, primarily through their unique combination of inherent hydrophilicity and high metallic conductivity [4], enabling facile processing in aqueous media. Their surface chemistry, characterized by abundant and tunable terminations (-O, -OH, -F) [6], offers a versatile platform for tailoring physicochemical properties to meet the demands of specific applications. Furthermore, their extensive compositional diversity (M_n+1_X_n_T_x_) provides access to a broad range of properties [10,11,12,13,14,15,16]. As research on 2D materials advances, MXenes are increasingly recognized for their potential in high-tech fields like spintronics, where magnetic and quantum effects are of critical importance [17].

Among the diverse family of MXenes, chromium-based MXenes stand out due to their magnetic properties. These materials not only offer new avenues for discovering intrinsic 2D magnetic materials but also provide a foundation for developing spintronic applications [18,19]. Unlike non-magnetic or weak magnetic MXenes, the magnetic properties of chromium-based MXenes can be significantly tuned by altering the surface functional groups. For instance, introducing different groups such as F, OH, or Cl to the Cr_2_C structure can yield a spectrum of magnetic states, ranging from antiferromagnetism to ferromagnetism [20]. This magnetic tunability arises from the interaction between the surface functional groups and the 3*d* orbitals of the chromium atoms, leading to changes in the electronic structure and magnetic moment. Recent studies have demonstrated that Cr_3_C_2_T_2_ (T = Cl, O, OH) monolayers can stably adsorb at least one layer of lithium atoms without incurring structural deformation, offering theoretical lithium storage capacities of 214, 253, and 250 mAh/g, respectively [21]. Given that the lithium storage capacity of 2D materials increases markedly with the number of adsorbed lithium layers, Cr_3_C_2_ and its functionalized derivatives (Cr_3_C_2_T_2_), show considerable promise as electrode materials for lithium-ion batteries [22].

Despite substantial advances in MXene research, especially in the domain of energy storage and catalysis [23], systematic investigations focused on the specific influence of surface functional groups on the electronic and magnetic properties of chromium-based MXenes remain limited. Currently, most efforts have been centered on theoretical calculations and preliminary experimental validations, while comprehensive studies exploring complex surface functionalization and doping strategies remain scarce [24]. Addressing these gaps necessitates rigorous investigations into the electronic structure and magnetic behavior of chromium-based MXenes, with particular emphasis on elucidating the regulatory effects of surface functional groups. To address this gap, this study investigates the modulation of magnetic ordering and Curie temperature through surface functionalization. Employing a computational framework that allows for free atomic relaxation while maintaining a fixed lattice, we capture the nuanced interplay between chemical terminations and magnetic interactions. Such studies are essential not only for bridging existing knowledge deficits but also for establishing a robust theoretical framework critical to the rational design of novel spintronic devices and functional materials.

In this study, density functional theory (DFT) was employed to investigate the magnetic properties, electronic structure, and Curie temperature of Cr_3_C_2_T_2_ (T = O, F, OH). The results reveal that surface functional groups play a crucial role in modulating the magnetic configuration, Curie temperature, and electronic structure of Cr_3_C_2_T_2_. Surface functionalization enables precise tuning of the Curie temperature from 461 K to 270 K, 272 K, and 337 K. This capability holds significant promise for tailoring materials to operate within specific magnetic refrigeration regimes, including cold chain logistics and waste heat recovery systems. The electronic structure transitions from a semi-metallic state exhibiting 100% spin polarization to metallic or semiconducting phases, introducing application-specific functionalities. The semi-metallic layer facilitates efficient spin injection and detection; the metallic layer acts as a low-resistance transport channel, and the semiconducting layer enables opto-spin conversion. This provides a new platform for developing next-generation room-temperature spintronics devices and magnetic–electronic–optical integrated systems. Calculations indicate that chemical functionalization can effectively control the magnetic easy axis direction: oxygen (O) functionalization increases the magnetic anisotropy energy (MAE) to +54.7 μeV, enhancing perpendicular magnetic anisotropy (PMA) and improving the thermal stability of MRAM storage units. In contrast, fluorine (F, −33.57 μeV) and hydroxyl (OH, −83.26 μeV) functionalizations induce strong in-plane magnetic anisotropy (IMA), suitable for magnonics. This on-demand tuning of PMA or IMA provides a powerful approach for designing spintronic devices with specific requirements. These findings provide valuable design principles for the development of MXene-based spintronic architectures and underscore the promise of chromium-based MXenes in advanced material applications.

## 2. Computing Method

All spin-polarized density functional theory (DFT) calculations were performed using the Vienna Ab-initio Simulation Package (VASP) [25,26]. The exchange–correlation interaction was treated using the Perdew–Burke–Ernzerhof (PBE) functional within the generalized gradient approximation (GGA), and the projector augmented wave (PAW) method was employed to describe the core–valence electron interactions [27,28]. To minimize interlayer interactions, a vacuum spacing greater than 15 Å was introduced along the *z*-axis. Ionic relaxation was performed until the forces on each atom were less than 0.02 eV/Å. The kinetic energy cutoff was set to 500 eV, and a 5 × 5 × 1 k-point mesh was applied using the Monkhorst–Pack scheme [29]. The on-site Coulomb interaction of the Cr 3*d* electrons was addressed using the GGA + *U* approach, with an effective *U* value of 3 eV, consistent with common practices in the literature [30,31,32]. Charge and magnetic moments on Cr ions were calculated by projecting the occupied wavefunctions onto spherical harmonics, which were non-zero within spheres centered on the Cr ions, as derived from the OUTCAR file of VASP. The MAE calculations explicitly account for spin-orbit coupling (SOC) through energy differences between in-plane (*E*_100_) and out-of-plane (*E*_001_) spin orientations. The phonon spectra were computed using the supercell approach in the PHONOPY software package [33].

## 3. Results and Discussion

### 3.1. Structural and Magnetic Properties

**Figure 1:**
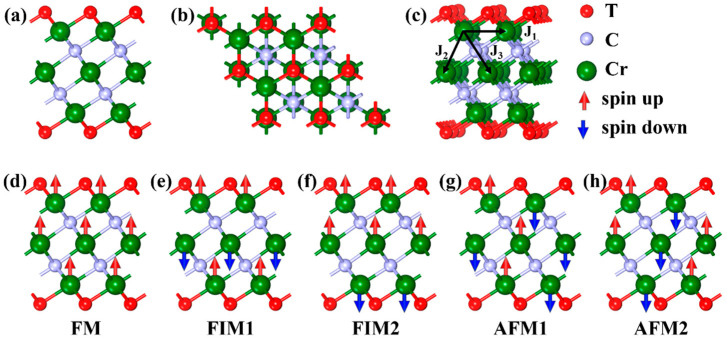
(**a**) The side and (**b**) top views of Cr_3_C_2_T_2_. (**c**) Schematic diagram of J_1_, J_2_, and J_3_ exchange coupling parameters in Cr_3_C_2_T_2_. (**d**–**h**) The five different magnetic configuration models, FM, FIM1, FIM2, AFM1, and AFM2, were considered in the calculation.

In this study, surface functionalization with O, F, and OH groups was modeled by chemically bonding these terminations at the hollow sites of Cr_3_C_2_, aligning with previous theoretical studies [21]. To capture the magnetic and structural variations induced by functionalization, a 2 × 2 supercell corresponding to the Cr_3_C_2_T_2_ stoichiometry (T = O, F, OH) was considered. Five distinct magnetic configurations were systematically analyzed: a ferromagnetic (FM), two distinct ferrimagnetic (FIM1 and FIM2), and two different antiferromagnetic arrangements (AFM1 and AFM2), as illustrated in Figure 1. This comprehensive treatment enables a detailed understanding of the influence of surface terminations on spin alignment and magnetic ground states in Cr-based MXenes.

The initial geometry optimization was performed on Cr_3_C_2_T_2_ without predefined magnetic ordering to determine equilibrium lattice parameters. Subsequently, calculations were carried out for all five magnetic states to identify the most energetically favorable configuration. The energy differences (∆*E*) relative to the FM state were calculated using the following expression:

(1) $$∆E=E_{O}-E_{FM}$$

where *E*_FM_ and *E*_O_ (representing E_FIM1_, E_FIM2_, E_AFM1_, and E_AFM2_) denote the total energies of the FM and other magnetic configurations of Cr_3_C_2_T_2_, respectively.

**Figure 2:**
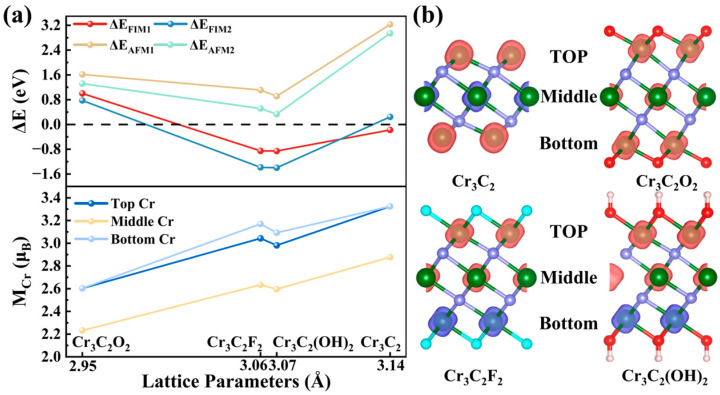
(**a**) Relative total energies of Cr_3_C_2_ and Cr_3_C_2_T_2_ (T = O, F, OH) systems under five magnetic configurations—ferromagnetic (FM), two ferrimagnetic (FIM1, FIM2), and two antiferromagnetic (AFM1, AFM2)—with the FM state used as the reference energy. Correlation between the magnetic moments of Cr atoms across different atomic layers and the corresponding lattice constants. (**b**) Spin density distribution of the most stable magnetic configurations of Cr_3_C_2_ and Cr_3_C_2_T_2_. The red and blue isosurfaces correspond to the spin-up and spin-down densities, respectively.

As shown in Figure 2a, the most stable configuration is F1M1 for Cr_3_C_2_, FM for Cr_3_C_2_O_2_, and FIM2 for both Cr_3_C_2_F_2_ and Cr_3_C_2_(OH)_2_. The spin density is predominantly localized on Cr atoms, as depicted in Figure 2b.

The magnetism in the Cr_3_C_2_T_2_ system arises predominantly from Cr atoms, whose magnetic moments correlate strongly with lattice constants at distinct terminations [34]. As shown in Figure 2a, based on the most stable magnetic ordering, the magnetic moments of Cr atoms vary systematically with lattice constant adjustments across terminations. Notably, outer-layer Cr atoms exhibit larger magnetic moments compared to the middle-layer atoms. The magnetic moments of middle-layer Cr atoms show a monotonic increase with the expansion of lattice constants, induced by the terminal group adsorption. This trend is largely mirrored by the outer-layer Cr atoms.

Intriguingly, despite the OH-terminated configuration (Figure 2a) having a larger lattice constant than the F-terminated system, the Cr atoms in the former display marginally smaller magnetic moments. This can be attributed to the higher electronegativity of fluorine, which induces stronger electron withdrawal. Consequently, this results in enhanced electron transfer in the F-terminated system, leading to a higher density of unpaired electrons on the outer-layer Cr atoms, amplifying their magnetic moments. Furthermore, increased interlayer spacing elongates Cr–Cr bonds, stabilizing unpaired electrons and reinforcing magnetic behavior.

Thus, our findings confirm that lattice constant variations, induced by different surface terminations, critically regulate the magnetic moments of Cr in Cr_3_C_2_T_2_. Expansion of the lattice constants increases intralayer bond lengths, weakens covalent interactions, and enhances the presence of unpaired electrons—all of which contribute to the enhancement of magnetic moments.

Cr_3_C_2_T_2_ adopts a top-to-bottom symmetrical geometry. Generally, the most energetically favorable magnetic configuration would also exhibit this symmetry. However, for Cr_3_C_2_F_2_ and Cr_3_C_2_(OH)_2_, the most stable FIM2 configuration is characterized by a top-to-bottom spin asymmetry. A detailed explanation is provided below to elucidate the underlying origin of this behavior.

**Figure 3:**
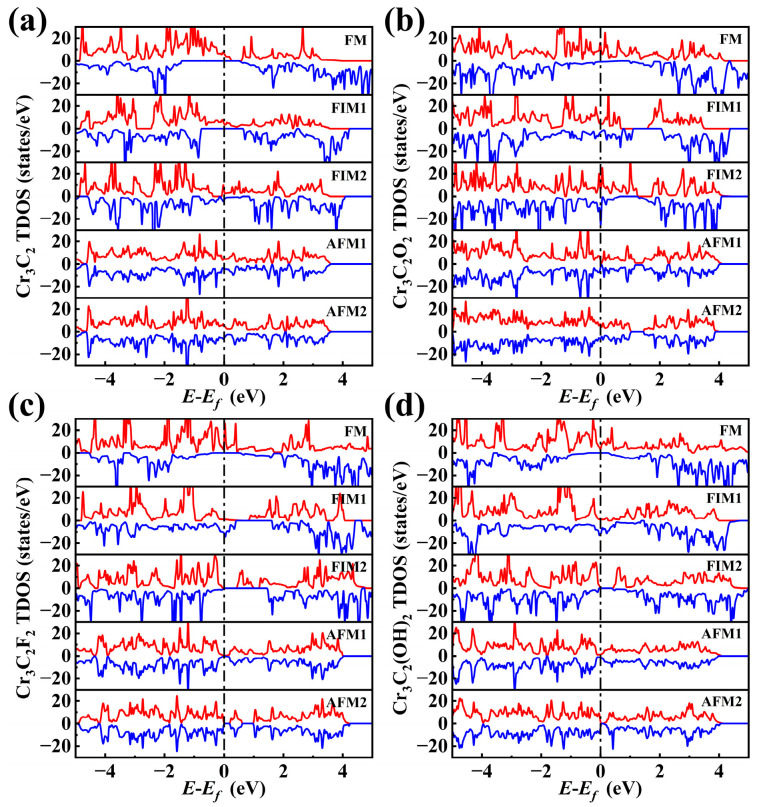
Total density of states (TDOSs) and bandgap of five magnetic configurations of Cr_3_C_2_T_2_. (**a**) Cr_3_C_2_; (**b**) Cr_3_C_2_O_2_; (**c**) Cr_3_C_2_F_2_; (**d**) Cr_3_C_2_(OH)_2_.

First, the density of states (DOSs) of Cr_3_C_2_T_2_ (T = O, F, OH) across the five magnetic configurations was analyzed, as shown in Figure 3 and summarized in Table 1. The stability of each configuration correlates with the spin-down DOSs above the Fermi level (*E* > 0). Although the electronic states above the Fermi level (*E* > 0) are unoccupied at absolute zero, they become accessible at finite temperatures via thermal excitation of the order of kT. Critically, in practical spintronic devices—such as magnetic tunnel junctions and spin valves—operating under non-equilibrium conditions, these states act as primary destinations for carrier injection and central mediators of spin transport. In ferromagnetic materials, the spin-resolved DOSs near the Fermi level (±eV) play an important role in governing (i) the magnitude of magnetoresistance effects, such as tunneling magnetoresistance (TMR) and giant magnetoresistance (GMR), (ii) the efficiency of spin injection, which depends on the electronic state matching at interfaces, and (iii) spin transfer torque (STT) efficiency, linked to the availability of final states for angular momentum exchange. Consequently, quantitative characterization of the DOSs at *E* > 0 is not only a theoretical pursuit but also an essential link between material magnetic order and device functionality—such as high-sensitivity sensing and low-power storage. For Cr_3_C_2_, both FM and FIM1 configurations display spin-down band gaps at *E* = 0. The spin-down band gap is 0.5 eV in the FM configuration and 0.65 eV in the FIM1 configuration. Our calculations confirm that the F1M1 configuration is the most stable for Cr_3_C_2_.

A similar trend is evident in Cr_3_C_2_F_2_ and Cr_3_C_2_(OH)_2_, which further supports this rule. In Cr_3_C_2_F_2_, the FIM2 configuration exhibits a spin-down band gap of 1.46 eV, exceeding the 0.53 eV gap in the FM configuration. Likewise, for Cr_3_C_2_(OH)_2_, the spin-down band gap of the FIM2 configuration is 0.37 eV, larger than the 0.24 eV of the FM configuration. These findings validate the FIM2 configuration as the most stable for both systems. Although Cr_3_C_2_O_2_ does not exhibit a band gap at the Fermi level in any magnetic configuration, the DOSs at *E* > 0 are substantially lower in the FM configuration, which is in agreement with the observed stability trend.

Second, the superior stability of the FIM2 configuration arises from optimized bond lengths and bond angles, which affect super-exchange interactions. In particular, the strength of antiferromagnetic interlayer coupling is dependent on the Cr–C–Cr bond angle.

Specifically, in Cr_3_C_2_F_2_, the Cr–C–Cr bond angle is approximately 177–178°in the FM, FIM1, AFM1, and AFM2 configurations. However, in the FIM2 configuration, the bond angle between first- and second-layer Cr atoms is 174.7°, while that between second- and third-layer Cr atoms is 179.1°. The reduction in bond angle weakens the antiferromagnetic super-exchange interaction between the first- and second-layer Cr atoms, favoring ferromagnetic coupling. Meanwhile, the second- and third-layer Cr atoms adopt a bond angle closer to 180°, enhancing the super-exchange interaction and favoring antiferromagnetic coupling in FIM2 configuration. The resulting antiferromagnetic ordering compensates for the increase in lattice energy caused by the bond length elongation by suppressing Coulomb repulsion between spin-polarized electrons. Similarly, Cr_3_C_2_(OH)_2_ follows the same trend.

Overall, the wide band gap and low unoccupied states of the FIM2 configuration reduce the excited-state energy. Concurrently, spin polarization limits high-energy state occupancy. Additionally, optimized bond angles significantly enhance antiferromagnetic coupling and mitigate Coulomb repulsion between spin-polarized electrons. These factors collectively account for the enhanced magnetic stability of the FIM2 state in Cr_3_C_2_F_2_ and Cr_3_C_2_(OH)_2_. The resulting strong in-plane anisotropy enables low-loss magnon waveguides and controllable magnetic domain-wall devices. These functionalities are essential for the development of energy-efficient spin-based information processing architectures. Thus, chemical functionalization serves as a bridge connecting the modulation of electronic structure to the practical implementation of spintronic technology.

### 3.2. Curie Temperature and Magnetic Anisotropy Energy

The Curie temperature (*T*_C_) represents the highest critical temperature at which magnetic materials retain stable ferromagnetism, a key property for spintronic device functionality. Heisenberg’s model was used to calculate the Curie temperature (*T*_C_) of the Cr_3_C_2_ and Cr_3_C_2_T_2_ (T = O, F, OH) systems. Initially, exchange coupling parameters (*J*_1_, *J*_2_, and *J*_3_) were calculated, where *J*_1_, *J*_2_, and *J*_3_ represent the nearest, next-nearest, and next-next-nearest neighbor exchange interactions, respectively, as shown in Figure 1c. The spin Hamiltonian, H_spin_, was calculated using the following:

(2) $$H_{spin}=-\sum_{i\neq j}\;J_{1}S_{i}.S_{j}-\sum_{k\neq l}\;J_{2}S_{k}.S_{l}-\sum_{m\neq n}\;J_{3}S_{m}.S_{n}$$

Here, S represents the net magnetic moment (3/2 μB) at the Cr site, while (i, j), (k, l), and (m, n) refer to Cr atom pairs at nearest, next-nearest, and next-next-nearest distances, respectively. Due to the variation in magnetic moments of Cr atoms across each layer in the Cr_3_C_2_ and Cr_3_C_2_T_2_ systems, a representative Cr atom from each layer was selected for formulation. As illustrated in Figure 1d, in the FM arrangement, all Cr ions exhibit parallel spin alignment, and the total system energy is given by Equation (2). The nearest, next-nearest, and next-next-nearest atomic interactions of the remaining magnetic configurations are shown in Table 2.

By mapping the DFT-derived total energies of FM, FIM1, FIM2, and AFM1 configurations onto the Heisenberg model, the following formula can be derived, expressed in terms of the exchange coupling parameters (*J*_1_, *J*_2_, and *J*_3_):

(3) $$E_{FM}=E_{0}-\left(36J_{1}+24J_{2}+24J_{3}\right)\left|S^{2}\right|$$

(4) $$E_{FIM1}=E_{0}-\left(36J_{1}-24J_{2}-24J_{3}\right)\left|S^{2}\right|$$

(5) $$E_{FIM2}=E_{0}-\left(36J_{1}\right)\left|S^{2}\right|$$

(6) $$E_{AFM1}=E_{0}-\left(-12J_{1}\right)\left|S^{2}\right|$$

(7) $$E_{AFM2}=E_{0}-\left(-12J_{1}+8J_{2}-24J_{3}\right)\left|S^{2}\right|$$

In this context, S denotes the net magnetic moment of the Cr atom, while *E*_FM_, *E*_FIM1_, *E*_FIM2_, *E*_AFM1_, and *E*_AFM2_ represent the total energy of the Cr_3_C_2_T_2_ system in various magnetic configurations. *E*_0_ is defined as the total energy of the system excluding exchange energy. The MAE was calculated by the following equation to investigate the spin directions:

(8) $$MAE=(E_{100}-E_{001})/N_{Cr}$$

Here, *E*_100_ and *E*_001_ represent the total energies of the Cr_3_C_2_T_2_ systems with the spins of the Cr atoms aligned along the [100] and [001] directions, respectively. *N*_Cr_ denotes the number of Cr atoms. A positive MAE indicates that the magnetic easy axis of Cr lies along the c-axis (out-of-plane), while a negative MAE implies an in-plane magnetic orientation.

As presented in Table 3, MAE varies with T. For T = O, the MAE is positive, with a significant enhancement of 54.7 μeV compared to pristine Cr_3_C_2_. In contrast, for T = F and OH, MAE becomes negative, marking a transition of the magnetic easy axis from out-of-plane to in-plane. This shift offers a viable strategy for tuning magnetic anisotropy in the Cr_3_C_2_T_2_ system.

**Figure 4:**
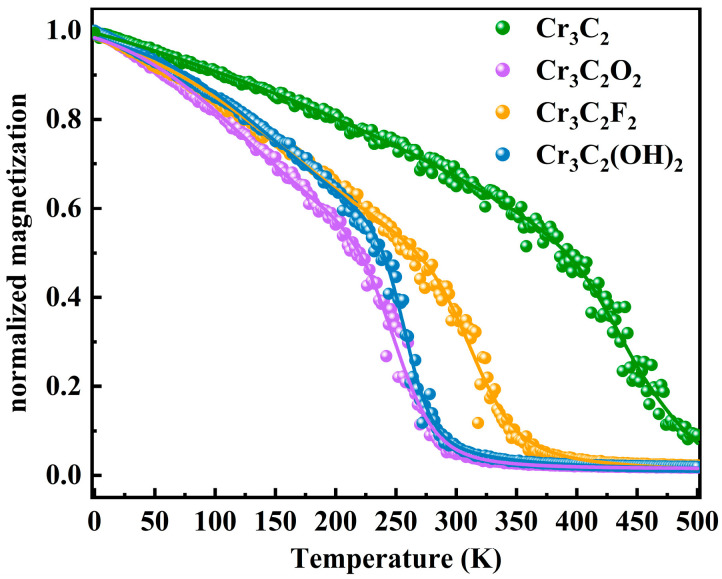
Magnetic and temperature profiles of Cr_3_C_2_ and Cr_3_C_2_T_2_ (T = O, F, OH).

The exchange coupling parameters (*J*_1_, *J*_2_, and *J*_3_), also presented in Table 2, change significantly with functionalization, directly impacting *T_C_*. *T_C_* values were estimated based on the Heisenberg model by using DFT-derived spin-exchange parameters. Monte Carlo (MC) simulations were also performed by the VAMPIRE package. As shown in Figure 4, the magnetic response as a function of temperature is highly tunable via surface chemistry. Specifically, Cr_3_C_2_ exhibits a *T_C_* of 461 K. The introduction of surface functional groups leads to a decrease in *T_C_* and enhances the temperature sensitivity of the magnetic moment. Notably, the *T_C_* of Cr_3_C_2_F_2_ is 337 K, which is still above room temperature and relatively high among 2D magnetic materials. Given theoretical reports of pristine Cr_3_C_2_ instability [35], the stabilized yet strongly magnetic nature of Cr_3_C_2_F_2_ underscores its practical relevance and application. Meanwhile, Cr_3_C_2_O_2_ and Cr_3_C_2_(OH)_2_ exhibit slightly lower *T_C_*, below room temperature, at 270 K and 272 K, respectively. Nonetheless, exploration of Cr-based MXenes continues to hold significant promise for spintronic applications.

### 3.3. Electronic Properties and Stability

**Figure 5:**
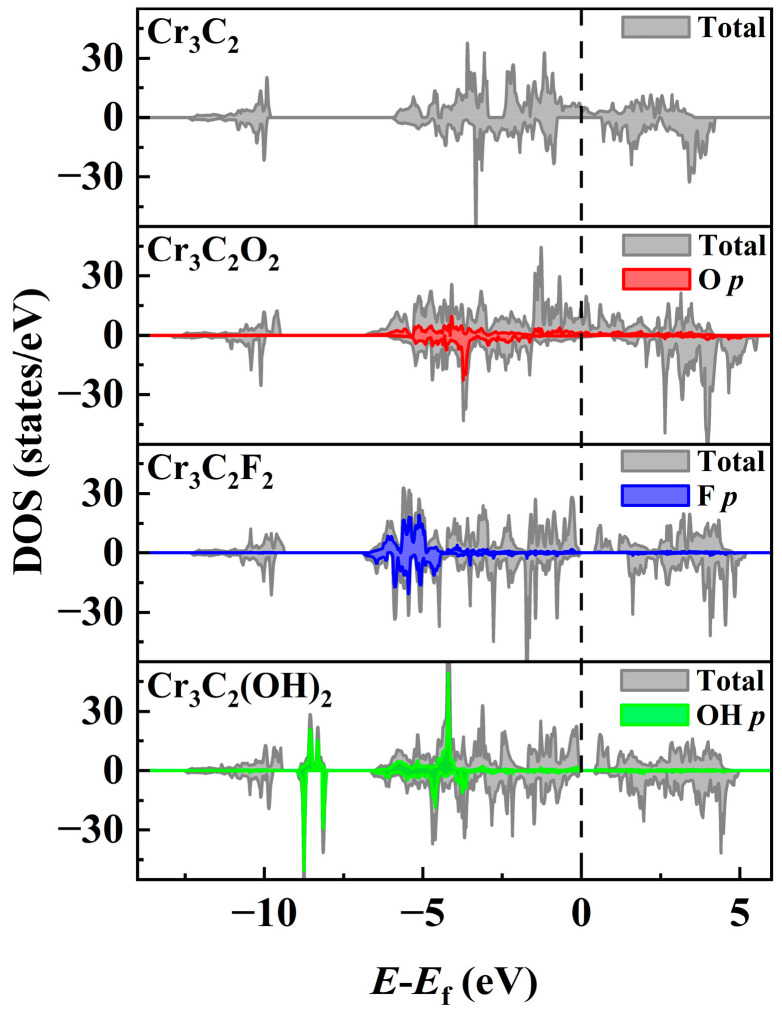
The total density of states for the Cr_3_C_2_ and Cr_3_C_2_T_2_ systems (T = O, F, OH) and the density of states contribution from T.

To assess the impact of surface termination on electronic properties, total DOSs calculations were performed for the Cr_3_C_2_T_2_ system. Notably, the introduction of different functional groups was found to profoundly alter the electronic behavior of these systems. As shown in Figure 5, pristine Cr_3_C_2_ exhibits half-metal characteristics, with a distinct band gap in the spin-down channel at the Fermi level, whereas Cr_3_C_2_O_2_ displays stronger metallic characteristics. This metallicity is attributed to the high electronegativity of oxygen, which redistributes the DOSs near the Fermi level, enhancing delocalized states.

In contrast, Cr_3_C_2_F_2_ and Cr_3_C_2_(OH)_2_ exhibit semiconducting properties, both featuring narrow band gaps. For Cr_3_C_2_F_2_, the strong electron-withdrawing nature of fluorine shifts the DOSs toward lower energy levels, increasing the band gap. In the case of Cr_3_C_2_(OH)_2_, the polarity of hydroxyl groups likely induces hydrogen bonding interactions or surface defect states, as evidenced by additional low-energy DOS peaks compared to pristine Cr_3_C_2_. A comparison of the spin-resolved DOSs across two functionalizations reveals that both exhibit significant spin asymmetry, characterized by marked differences between the spin-up and spin-down channels in the vicinity of the Fermi level. This inherent spin polarization is a defining attribute of the magnetic behavior in these systems and is integral to their potential use in spintronic applications, where spin-selective transport is a prerequisite.

**Figure 6:**
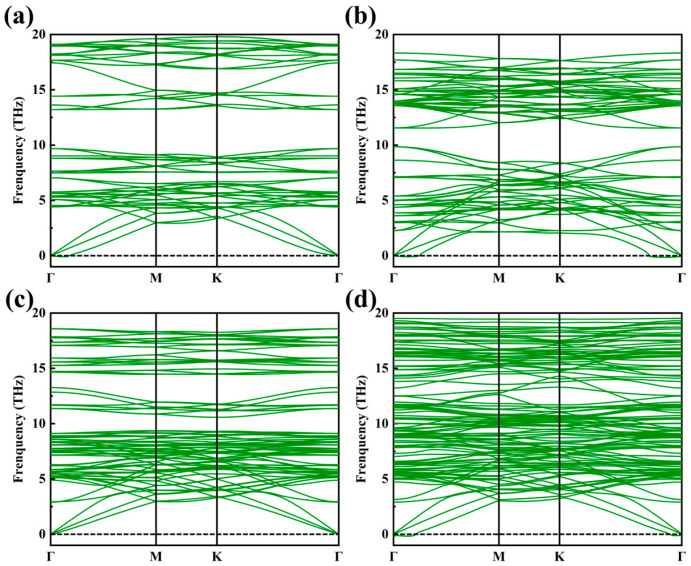
Phonon dispersion curve of Cr_3_C_2_T_2_ in the most stable magnetic ground state. (**a**) Cr_3_C_2_; (**b**) Cr_3_C_2_O_2_; (**c**) Cr_3_C_2_F_2_; (**d**) Cr_3_C_2_(OH)_2_.

To evaluate the thermodynamic stability of these compounds, we performed phonon spectrum calculations using the DFPT method. Figure 6 shows the phonon band structure of Cr_3_C_2_T_2_. Cr_3_C_2_F_2_ has no imaginary frequencies, and the imaginary frequencies of the other structures do not exceed 5 cm^−1^, indicating that these four structures are dynamically stable.

Tuning the electronic structure is essential in the design of spintronic devices. Pristine Cr_3_C_2_, with its 100% spin polarization and semi-metallic nature, holds significant potential for magnetic storage, spin logic devices, and quantum technologies. The introduction of oxygen functionalized Cr_3_C_2_O_2_, with enhanced conductivity and carrier density due to its metallic nature, is promising for energy storage and conversion applications [36]. Moreover, Cr_3_C_2_F_2_ and Cr_3_C_2_(OH)_2_, as semiconducting materials, exhibit extremely narrow band gaps, positioning them as potential materials for high-speed electronics, optoelectronics, and thermoelectric devices [37]. Although surface termination with fluorine or hydroxyl groups can enhance the stability of Cr_3_C_2_, it comes at the cost of substantially sacrificing spin polarization, effectively eliminating half-metallic behavior. As a consequence, these functionalized configurations become unsuitable for applications requiring high spin selectivity, such as spin filters and spin injectors. This trade-off highlights the importance of balancing structural robustness with magnetic and electronic performance in the design of functional MXene-based spintronic devices. Based on the performance of the Cr_3_C_2_T_2_ system in various aspects, as shown in Table 4, the most suitable applications for each surface functionalization system are listed and explained.

## 4. Conclusions

In this study, a spin-polarized DFT method was employed to systematically investigate the effects of surface functionalization on the electronic and magnetic properties of Cr_3_C_2_T_2_ (T = O, OH, F). The stable magnetic configurations of Cr_3_C_2_T_2_ were determined, revealing an asymmetric ferrimagnetic ground state in certain cases, supported by DOSs and lattice relaxation. The tunable antiferromagnetic behavior and pronounced spin asymmetry revealed in this study provide a key materials physics foundation for advancing next-generation spintronic technologies. These findings offer critical insights for the design of high-speed, high-density, and energy-efficient magnetic memory and spin-based logic devices. Additionally, we demonstrated that surface termination significantly affects magnetic properties, including Curie temperature and magnetic anisotropy. These results highlight the potential for surface engineering as a tool to tailor magnetic ordering and orientation in chromium-based MXenes. Furthermore, a comprehensive analysis of electronic structures revealed that oxygen functionalization induces metallic behavior, whereas fluorination and hydroxylation lead to semiconducting states with narrow band gaps. It should be noted that actual MXenes may have a disordered distribution of functional groups or vacancy defects, which may weaken but not fundamentally alter the dominant effect of intrinsic functional groups. These modulated properties offer a pathway to optimize Cr_3_C_2_T_2_ materials for spintronic, optoelectronic, and thermoelectric applications.

## Figures and Tables

**Table 1:** Spin-down band gap (*E* > 0) and bond angles between the first and second layers, as well as between the second and third layers of Cr-C-Cr of Cr_3_C_2_ and Cr_3_C_2_T_2_ system. Bold represents the most stable magnetic state.

	Ground State	Spin-Down Band Gap (*E* > 0) (eV)	∠Cr_1_-C_1_-Cr_2_ (°)	∠Cr_2_-C_2_-Cr_3_ (°)
Cr_3_C_2_	FM	0.50	170.77	170.77
	**FIM1**	**0.65**	170.13	170.13
	FIM2	…	168.26	174.99
	AFM1	…	170.84	170.84
	AFM2	…	174.26	169.77
Cr_3_C_2_O_2_	**FM**	…	176.30	176.30
	FIM1	…	175.86	175.86
	FIM2	…	176.93	175.38
	AFM1	…	174.73	174.73
	AFM2	…	176.00	176.58
Cr_3_C_2_F_2_	FM	0.53	177.42	177.42
	FIM1	…	178.29	178.29
	**FIM2**	**1.46**	174.66	179.05
	AFM1	…	176.91	177.34
	AFM2	0.18	177.87	176.78
Cr_3_C_2_(OH)_2_	FM	0.25	178.01	177.73
	FIM1	…	179.07	178.86
	**FIM2**	**0.37**	174.67	179.67
	AFM1	…	177.77	177.50
	AFM2	0.07	177.94	177.66

**Table 2:** The number of nearest, next-nearest, and next-next-nearest interactions between the five magnetic configurations FM, FIM1, FIM2, AFM1, and AFM2.

Ground State	Nearest			Next-Nearest			Next-Next-Nearest		
	Upper Layer	Middle Layer	Bottom Layer	Upper Layer	Middle Layer	Bottom Layer	Upper Layer	Middle Layer	Bottom Layer
FM	6/0	6/0	6/0	3/0	6/0	3/0	3/0	6/0	3/0
FIM1	6/0	6/0	6/0	0/3	0/6	0/3	0/3	0/6	0/3
FIM2	6/0	6/0	6/0	3/0	3/3	0/3	3/0	3/3	0/3
AFM1	2/4	2/4	2/4	1/2	3/3	2/1	3/0	3/3	0/3
AFM2	2/4	2/4	2/4	2/1	4/2	2/1	0/3	0/6	0/3

**Table 3:** *J*_1_, *J*_2_, and *J*_3_, MAE, and *T_C_* of Cr_3_C_2_T_2_ systems.

	Ground State	*M* (µ_B_)	Lattice Parameters (Å)	MAE (μeV)	*J*_1_ (meV)	*J*_2_ (meV)	*J*_3_ (meV)	*T_C_* (K)
Cr_3_C_2_	FIM1	13.71	3.14	22.01	30.7	2.8	−4.4	461
Cr_3_C_2_O_2_	FM	25.59	2.95	54.70	10.3	11.1	−1.8	270
Cr_3_C_2_F_2_	FIM2	8.47	3.06	−33.57	14.2	−14.2	6.3	337
Cr_3_C_2_(OH)_2_	FIM2	8.43	3.07	−83.26	12.4	2.1	−10	272

**Table 4:** Applications of the Cr_3_C_2_T_2_ system.

	Material Properties	Optimal Device Architecture	Device Advantages
Cr_3_C_2_	Perpendicular magnetic anisotropy (MAE = +22.01 μ eV) Half-metal High Curie temperature (*T*_C_ = 461 K)	Spin Valve	Magnetic anisotropy: simplified control of magnetic moment direction; High spin polarization: realize efficient spin current transmission; High operating temperature: *T*_C_ of 461 K ensures the stability of the device in high-temperature environments
Cr_3_C_2_O_2_	Strong perpendicular magnetic anisotropy (MAE = +54.7 μ eV) Metallic High Curie temperature (*T*_C_ = 270 K)	MRAM storage cell	High vertical anisotropy: improves storage density and thermal stability; Metallic: low contact resistance; *T*_C_ Approaching room temperature: Suitable for embedded storage
Cr_3_C_2_F_2_	In-plane magnetic anisotropy (MAE = −33.57 μ eV) Narrow bandgap semiconductor *T*_C_ = 337 K	Spin wave guide/Spin logic device Spin-FET channel	In-plane anisotropy: supports low-loss spin wave propagation; Semiconductor properties: gate voltage regulation of spin transport; Higher *T*_C_: room temperature operation
Cr_3_C_2_(OH)_2_	Strong in-plane magnetic anisotropy (MAE = −83.26 μ eV) Narrow bandgap Flexible interface compatibility	Flexible Spin Sensor Low-Power Spin-FET	High in-plane anisotropy: high magnetic resistance response sensitivity; Narrow bandgap: low driving voltage; OH terminal hydrophilicity: adaptation to flexible substrates

## Data Availability

The original contributions presented in this study are included in the article. Further inquiries can be directed to the corresponding authors.
